# Physiology-guided bilateral pulmonary artery banding in high-risk neonates: early hemodynamic and perfusion outcomes

**DOI:** 10.3389/fcvm.2026.1803385

**Published:** 2026-05-04

**Authors:** Ahmet Kuddusi İrdem, Hasan Balık, Yiğit Kılıç, Peren Sarız Dinç

**Affiliations:** 1 Department of Pediatric Cardiovascular Surgery, Ankara Bilkent City Hospital, Ankara, Türkiye; 2 Department of Pediatric Cardiology, Diyarbakir SBU Gazi Yasargil Training and Research Hospital, Diyarbakir, Türkiye; 3 Department of Pediatric Cardiovascular Surgery, Diyarbakir SBU Gazi Yasargil Training and Research Hospital, Diyarbakir, Türkiye; 4 Department of Pediatrics, Ankara Bilkent City Hospital, Ankara, Türkiye

**Keywords:** bilateral pulmonary artery banding, ductal-dependent circulation, end-tidal carbon dioxide, high-risk neonates, near-infrared spectroscopy, physiology-guided pulmonary artery banding, pulmonary blood flow

## Abstract

**Background:**

Balancing pulmonary and systemic blood flow remains one of the most critical challenges in the management of high-risk neonates with ductal-dependent circulation, particularly in the presence of low birth weight, sepsis, shock, restrictive interatrial communication, or complex congenital cardiac anatomy. Bilateral pulmonary artery banding (biPAB) is frequently employed as an initial stabilizing strategy to control pulmonary overcirculation; however, optimal band tightness remains largely experience-based, and uniform band sizing may be associated with variable physiological responses. This study evaluated whether a physiology-guided, individualized approach to biPAB is associated with more favorable early hemodynamic and perfusion profiles compared with a conventional uniform banding strategy.

**Methods:**

This retrospective, two-center cohort study included critically ill neonates undergoing emergency bilateral pulmonary artery banding for ductal-dependent systemic and/or coronary circulation. Patients were managed using either a conventional uniform banding technique or a physiology-guided strategy based on body weight–adjusted branch pulmonary artery z-scores targeting an approximate −2 z-score diameter. Early postoperative assessment included serial measurements of systemic arterial pressure, arterial oxygen saturation, end-tidal carbon dioxide (etCO₂), Doppler-derived pulmonary artery flow velocities, and regional cerebral and somatic oxygenation assessed by near-infrared spectroscopy.

**Results:**

A total of 44 high-risk neonates underwent bilateral pulmonary artery banding (conventional banding, *n* = 20; physiology-guided banding, *n* = 24). Both strategies were associated with increases in systemic arterial pressure. Compared with the conventional group, the physiology-guided group demonstrated greater and more consistent reductions in etCO₂, together with higher Doppler-derived pulmonary artery flow velocities. Postoperative arterial oxygen saturation was lower in the physiology-guided group, while differences between arterial oxygen saturation and regional cerebral and somatic oxygenation were smaller. Absolute regional oxygenation values remained stable in both groups. Early postoperative and interstage mortality did not differ between strategies.

**Conclusions:**

In high-risk neonates with ductal-dependent circulation, a physiology-guided bilateral pulmonary artery banding strategy based on weight-adjusted pulmonary artery z-scores was associated with more predictable modulation of pulmonary blood flow and more consistent early hemodynamic and perfusion profiles, without an observed increase in early or interstage mortality. These findings support the feasibility of integrating individualized, physiology-driven principles and multimodal physiological monitoring into neonatal pulmonary artery banding strategies.

## Introduction

The management of high-risk neonates with ductal-dependent systemic and/or coronary circulation represents one of the most complex challenges in contemporary congenital cardiac surgery (1, 2). In these patients, survival depends on achieving a delicate balance between pulmonary and systemic blood flow during a period of profound vulnerability. Even minor perturbations in vascular resistance, ventricular loading conditions, or oxygen delivery may result in rapid clinical deterioration, particularly in neonates with low birth weight, sepsis, shock, restrictive interatrial communication, or complex congenital cardiac anatomy (3–6).

Pulmonary overcirculation is a common and potentially devastating problem in ductal-dependent neonates. Excessive pulmonary blood flow may lead to systemic hypoperfusion, metabolic acidosis, renal dysfunction, and impaired cerebral oxygen delivery, while also increasing the risk of pulmonary edema and respiratory failure (7–10). Conversely, excessive restriction of pulmonary blood flow can result in severe hypoxemia and hemodynamic instability. Achieving an optimal balance is therefore critical but inherently difficult, especially during the early neonatal period when pulmonary vascular resistance is rapidly evolving. These challenges highlight the need for strategies that integrate anatomical planning with real-time physiological feedback rather than relying solely on fixed anatomical targets.

Bilateral pulmonary artery banding (biPAB) has emerged as an important initial stabilizing strategy in critically ill neonates who are poor candidates for immediate definitive surgical repair (7–10). By restricting pulmonary blood flow at the branch pulmonary artery level, biPAB aims to reduce pulmonary overcirculation, improve systemic perfusion pressure, and allow time for physiological recovery, growth, and reassessment of surgical options. This strategy is commonly employed in neonates with hypoplastic left heart syndrome, severe aortic arch hypoplasia, borderline ventricular morphology, and other forms of ductal-dependent physiology (11–15).

Despite its widespread use, biPAB remains a physiologically demanding intervention with a narrow therapeutic window. Optimal band tightness is crucial, yet remains poorly standardized. In many centers, uniform band sizing based on fixed circumferential measurements is applied across a broad spectrum of patient sizes and pulmonary artery dimensions (16, 17). In small neonates, however, millimeter-level differences in band circumference may translate into disproportionately large changes in pulmonary blood flow, resulting in either inadequate restriction or excessive obstruction, as predicted by fundamental flow–radius relationships (18).

The limitations of uniform banding strategies have prompted growing interest in more individualized approaches to pulmonary artery banding. Weight-adjusted pulmonary artery z-scores provide a normalized framework for estimating target vessel dimensions based on patient size, potentially allowing for more reproducible anatomical and hemodynamic effects (19–21). However, anatomical calibration alone may be insufficient, as the physiological consequences of pulmonary blood flow restriction are influenced by dynamic factors including pulmonary vascular resistance, ventricular function, ductal flow patterns, and systemic vascular tone (16, 17).

Recent advances in perioperative physiological monitoring have expanded the clinician's ability to assess pulmonary blood flow and end-organ perfusion in real time. End-tidal carbon dioxide (etCO₂) has been increasingly recognized as a surrogate marker of pulmonary blood flow in the absence of significant ventilation–perfusion mismatch (22–24), while near-infrared spectroscopy (NIRS) provides continuous, noninvasive assessment of regional cerebral and somatic oxygenation (20, 25). Together, these modalities offer complementary insights into the balance between pulmonary and systemic circulation that are not captured by arterial oxygen saturation or blood pressure alone.

Integrating anatomical planning with physiological feedback may therefore offer a more robust framework for optimizing pulmonary artery banding. A physiology-guided approach that incorporates patient-specific pulmonary artery dimensions alongside real-time hemodynamic and perfusion monitoring has the potential to reduce interpatient variability, improve reproducibility, and enhance early postoperative stability (16, 19).

Accordingly, the present study evaluated early hemodynamic and perfusion-related outcomes following bilateral pulmonary artery banding in a cohort of critically ill, high-risk neonates. We compared outcomes following a conventional uniform banding strategy with those of a physiology-guided approach based on body weight–adjusted pulmonary artery z-scores and multimodal physiological monitoring. Specifically, we explored whether an individualized, physiology-guided banding strategy was associated with more predictable modulation of pulmonary blood flow and more consistent early hemodynamic and perfusion profiles, without an apparent compromise in early clinical outcomes.

### Patients and methods

#### Study design and ethical approval

This retrospective, two-center cohort study was conducted in accordance with the principles of the Declaration of Helsinki. Approval was obtained from the institutional review boards of both participating centers prior to data collection. Written informed consent for surgical intervention and for the use of anonymized clinical data for research purposes was obtained from the parents or legal guardians of all patients.

Clinical, operative, and postoperative data were retrospectively extracted from institutional electronic medical records, anesthesia charts, echocardiography reports, and intensive care unit databases. Data collection focused on perioperative physiological parameters, hemodynamic measurements, and early clinical outcomes following bilateral pulmonary artery banding (biPAB).

#### Patient population and inclusion criteria

Critically ill neonates with ductal-dependent systemic and/or coronary circulation who underwent emergency bilateral pulmonary artery banding between January 2018 and January 2026 were eligible for inclusion. Ductal dependency was defined as reliance on patent ductus arteriosus flow to maintain adequate systemic or coronary perfusion.

Patients were considered high risk if one or more of the following criteria were present: low birth weight (≤2,500 g), preoperative shock requiring vasoactive support, documented sepsis, evidence of end-organ dysfunction (including renal or hepatic impairment), restrictive or intact interatrial communication, or complex congenital cardiac anatomy such as hypoplastic left heart syndrome, critical aortic arch hypoplasia, or borderline ventricular morphology.

Patients requiring extracorporeal membrane oxygenation support or peritoneal dialysis either before or after biPAB were excluded from comparative analysis. This exclusion was intended to reduce confounding from extreme physiological instability and to allow a more homogeneous evaluation of early physiological responses to pulmonary blood flow modulation; however, it may limit generalizability to the most critically unstable neonatal population.

#### Study groups and banding strategy

Patients were stratified into two groups according to the pulmonary artery banding strategy employed during the study period.

In the conventional banding group, band sizing was determined according to standardized institutional practice using fixed PTFE band diameters. Specifically, a 3.0-mm band was used for neonates weighing ≤3 kg, while a 3.5-mm band was used for those weighing >3 kg, without systematic adjustment based on pulmonary artery dimensions, z-scores, or physiological parameters beyond general intraoperative clinical judgment.

The physiology-guided group underwent individualized banding using branch pulmonary artery z-score targets, with body weight serving as a practical clinical reference during operative planning.

Pulmonary artery z-score estimation was performed using a body surface area (BSA)-based reference system, with BSA calculated according to the Haycock formula. Target branch pulmonary artery diameters corresponding to approximately a −2 z-score were used as an initial anatomical reference. Representative target pulmonary artery diameters and corresponding band circumferences based on weight-derived z-score estimates are presented in Table 1. These target diameters were subsequently translated into estimated band circumferences using the formula C = 2πr.

**Table 1:** Representative target pulmonary artery diameters and corresponding band circumferences based on weight-derived z-score estimates, compared with conventional band sizing.

Weight (g)	Target RPA diameter (mm)	Radius (mm)	Band circumference (mm)	Conventional band diameter (mm)	Conventional circumference (mm)
2000	2.75	1.375	8.64	3.00	9.42
2250	2.80	1.40	8.80	3.00	9.42
2500	2.90	1.45	9.11	3.00	9.42
2750	3.00	1.50	9.42	3.00	9.42
3000	3.10	1.55	9.73	3.00	9.42

Band circumference was calculated using the formula *C* = 2*πr*.

Importantly, this anatomical target was not considered absolute. Final band tightening was refined intraoperatively according to physiological responses. End-tidal CO₂ was used as the primary surrogate marker of pulmonary blood flow and served as the principal driver of intraoperative band adjustment, interpreted in conjunction with systemic arterial pressure, near-infrared spectroscopy (NIRS), and Doppler-derived flow characteristics (26, 27). In selected cases, physiological optimization required modest additional tightening beyond the initial −2 z-score target, typically corresponding to approximate z-score values in the range of −2.3 to −2.4.

Actual achieved band diameters or circumferences were not systematically recorded in a standardized manner, and therefore direct quantitative comparison of final band tightness between groups was not possible. Therefore, comparisons between strategies are based on intended anatomical targets and observed physiological responses rather than direct measurements of final band dimensions.

**Figure 1:**
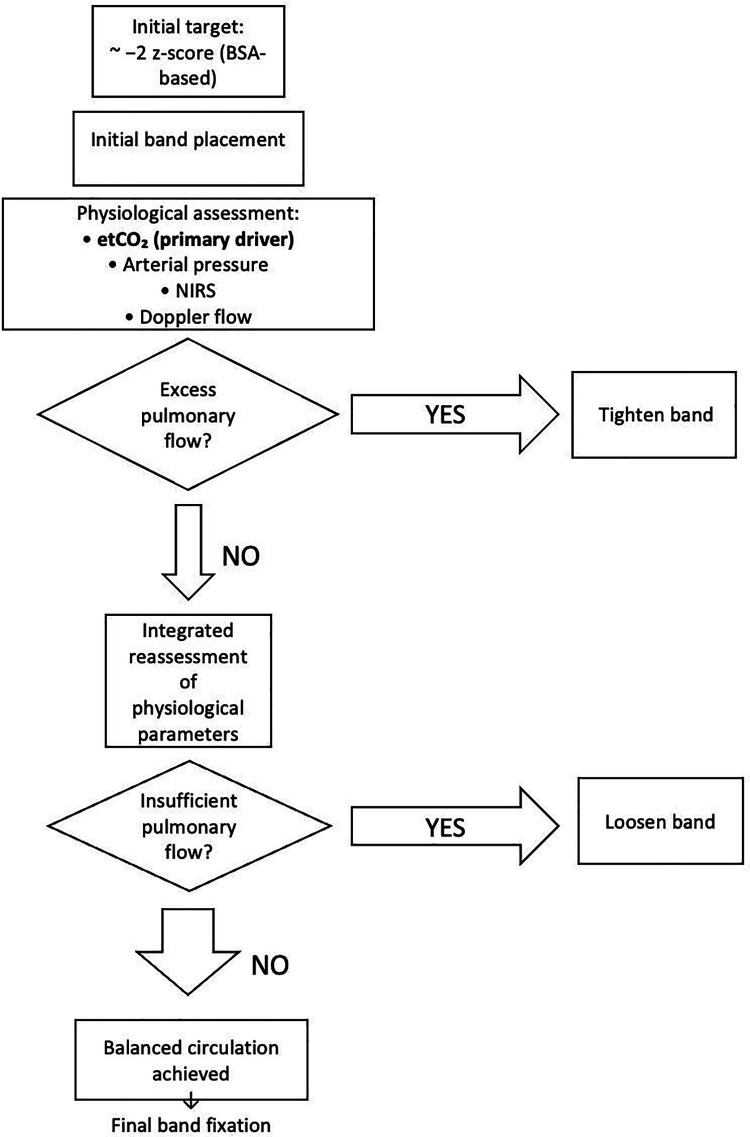
Decision-making algorithm for physiology-guided pulmonary artery band adjustment based on multimodal physiological parameters.

The decision-making algorithm for physiology-guided band adjustment is shown in Figure 1.

**Figure 2:**
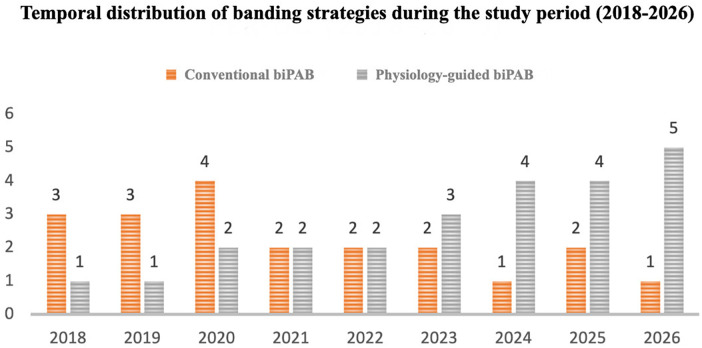
Temporal distribution of conventional and physiology-guided bilateral pulmonary artery banding (biPAB) strategies during the study period (2018–2026), demonstrating partial overlap between the two approaches.

The physiology-guided strategy was introduced during the study period by a single surgeon and was progressively adopted as experience with the technique increased. Although the number of physiology-guided cases was initially smaller, both banding strategies were applied during overlapping time intervals rather than representing two distinct eras of practice. The increasing use of the physiology-guided approach therefore reflects a gradual institutional adoption of the technique together with greater integration of multimodal physiological monitoring into intraoperative decision-making. The temporal distribution and progressive adoption of conventional and physiology-guided banding strategies across the study period are illustrated in Figure 2

### Surgical technique

All procedures were performed via median sternotomy under general anesthesia. Following systemic heparinization, bilateral pulmonary artery banding was performed without cardiopulmonary bypass (28). Banding was initiated on the right pulmonary artery followed by the left pulmonary artery to allow sequential assessment of hemodynamic and physiological responses.

PTFE material was used for all bands. In the physiology-guided group, band tightening was performed incrementally with continuous assessment of physiological parameters to achieve target hemodynamic goals while avoiding excessive restriction. Band position and adequacy were confirmed visually and by intraoperative assessment (27).

### Physiological monitoring and rationale

Continuous invasive arterial blood pressure monitoring was employed in all patients. End-tidal carbon dioxide (etCO₂) monitoring was used as a surrogate marker of pulmonary blood flow, based on the principle that, in the absence of significant ventilation–perfusion mismatch, etCO₂ primarily reflects pulmonary perfusion and cardiac output.

Near-infrared spectroscopy (NIRS) was used to continuously monitor regional cerebral and somatic oxygen saturation throughout the perioperative period. Sensors were positioned according to manufacturer recommendations, and values were recorded continuously. Predefined perioperative time points were selected for analysis to ensure consistency across patients.

Interpretation of NIRS data focused on the relationship between arterial oxygen saturation (SaO₂) and regional tissue oxygenation (rSO₂), rather than on absolute rSO₂ values alone. Differences between SaO₂ and rSO₂ were used as a relative indicator of the balance between systemic oxygen delivery and tissue-level oxygen utilization.

### Physiological rationale for the guided strategy

Pulmonary blood flow regulation in ductal-dependent neonates is governed by highly nonlinear physiological relationships. Rapid postnatal changes in pulmonary vascular resistance, combined with immature ventricular compliance and variable ductal flow patterns, render fixed anatomical strategies particularly vulnerable to over- or under-correction.

Weight-adjusted pulmonary artery z-scores were selected as the anatomical framework for individualized band sizing in order to normalize target vessel dimensions across a heterogeneous population. A target of approximately −2 z-score diameter was chosen as a standardized starting point to reduce pulmonary overcirculation while avoiding critical obstruction.

End-tidal carbon dioxide trends were interpreted as dynamic indicators of pulmonary blood flow and assessed in conjunction with systemic arterial pressure and echocardiographic findings to minimize misinterpretation due to transient ventilatory or cardiac output changes. Near-infrared spectroscopy provided complementary information regarding systemic and regional perfusion. A widening SaO₂–rSO₂ gradient was interpreted as a marker of impaired oxygen delivery relative to tissue demand, whereas a narrowing gradient suggested improved coupling between systemic perfusion and end-organ oxygen utilization.

The integration of anatomical planning with multimodal physiological monitoring allowed band adjustments to be guided by both structural targets and functional responses. The conceptual impact of small differences in band circumference on pulmonary blood flow, as illustrated using Poiseuille's law, is presented as a conceptual framework rather than as a predictive physiological model.

### Postoperative management and assessment

Postoperative management was standardized across centers and included mechanical ventilation, vasoactive support as required, and continuous physiological monitoring. Serial measurements of systemic arterial pressure, arterial oxygen saturation, etCO₂, and NIRS-derived cerebral and somatic oxygenation were obtained during the early postoperative period.

Echocardiographic assessment was performed to evaluate pulmonary artery band adequacy, with Doppler-derived flow velocities measured across both pulmonary artery bands. Renal function was assessed using serial serum creatinine measurements, particularly in patients with preoperative renal dysfunction.

### Outcome measures

Primary outcomes included early hemodynamic and perfusion-related parameters following biPAB, including changes in systemic arterial pressure, etCO₂, pulmonary artery flow velocities, and regional tissue oxygenation. Secondary outcomes included early postoperative mortality, interstage mortality, and progression to subsequent staged palliation or definitive repair.

### Statistical analysis

Statistical analyses were performed using IBM SPSS Statistics (IBM Corp., Armonk, NY, USA). Continuous variables were expressed as mean ± standard deviation or median with interquartile range, depending on data distribution. Categorical variables were presented as counts and percentages.

Group comparisons were performed using the independent-samples *t*-test or Mann–Whitney *U*-test for continuous variables and the chi-square test or Fisher's exact test for categorical variables, as appropriate. Given the retrospective and exploratory nature of the study, analyses were intended to support descriptive interpretation rather than formal hypothesis testing. No adjustment for multiple comparisons was applied. All statistical tests were two-sided, with a *p*-value < 0.05 considered indicative of statistical significance.

## Results

### Patient characteristics

**Figure 3:**
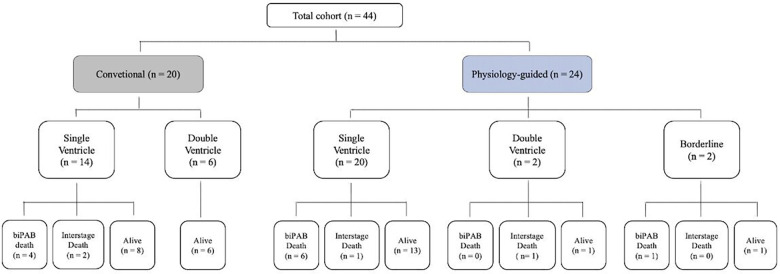
Flow diagram of the study cohort showing patient distribution according to banding strategy, ventricular physiology, and early outcomes, including perioperative mortality, interstage mortality, and survival status.

During the study period, 54 neonates underwent emergency bilateral pulmonary artery banding. After exclusion of patients requiring extracorporeal membrane oxygenation support or peritoneal dialysis, 44 high-risk neonates were included in the final analysis. Of these, 20 patients underwent conventional uniform banding, while 24 patients were managed using the physiology-guided banding strategy. The distribution of patients according to banding strategy, ventricular morphology, and clinical outcomes is summarized in Figure 3.

Underlying cardiac diagnoses and anatomical classification of the study population, along with their distribution across the conventional and physiology-guided groups, are summarized in Table 2. The distribution of underlying cardiac anatomy included single-ventricle physiology, double-ventricle physiology, and borderline ventricular morphology (29). Preoperative physiological parameters were broadly comparable between groups; however, the physiology-guided group included a higher proportion of low-birth-weight neonates, reflecting the later adoption of this strategy in smaller and more physiologically fragile patients. Operative time was longer in the physiology-guided group compared with the conventional group [median 56 [52–61] min vs. 46 [41–55] min].

**Table 2:** Underlying cardiac diagnoses and anatomical classification of neonates undergoing bilateral pulmonary artery banding (*n* = 44).

Cardiac diagnosis	Total (*n* = 44)	Conventional (*n* = 20)	Physiology-guided (*n* = 24)
Single ventricle (*n* = 34)	34	14	20
HLHS (*n* = 28)	28	11	17
AA-MA	16	7	9
AA-MS	1	1	0
AS-MA	5	1	4
AS-MS	4	1	3
AA with unbalanced CAVCD	2	1	1
Single ventricle type large VSD, arch hypoplasia	2	1	1
DILV-TGA, arch hypoplasia	4	2	2
Double ventricle (*n* = 8)	8	6	2
TGA-VSD, arch hypoplasia	2	2	0
CAVCD, arch hypoplasia	3	2	1
Bicuspid AV, arch hypoplasia	3	2	1
Borderline hypoplastic ventricle (*n* = 2)			
LV hypoplasia, arch hypoplasia	2	0	2

AA, aortic atresia; AS, aortic stenosis; AV, aortic valve; BAV, bicuspid aortic valve; CAVCD, complete atrioventricular canal defect; DILV, double inlet left ventricle; HLHS, hypoplastic left heart syndrome; LV, left ventricle; MA, mitral atresia; MS, mitral stenosis; TGA, transposition of the great arteries; VSD, ventricular septal defect.

Values represent the number of patients in each.

### Global hemodynamic response to bilateral pulmonary artery banding

Across the entire cohort, bilateral pulmonary artery banding was associated with an increase in systemic arterial pressure. Systolic, diastolic, and mean arterial pressures increased following banding in both groups.

Systemic hemodynamic and physiological parameters before and after bilateral pulmonary artery banding are presented in Table 3, while detailed baseline and early postoperative changes according to banding strategy are summarized in Table 4. No statistically significant between-group differences were observed in baseline arterial blood gas parameters or lactate levels.

**Table 3:** Systemic hemodynamic and physiological parameters before and after bilateral pulmonary artery banding according to banding strategy.

Hemodynamic parameters	Pre-biPAB average (after induction of anesthesia)			Early postoperative measurements (day 0 and day 3)		
	Conventional biPAB group	Physiology-guided biPAB group	*p*-value	Conventional biPAB group	Physiology-guided biPAB group	*p*-value
SAP (mmHg)	46.7 ± 4.7	45.2 ± 4.6	0.285*	58.5 ± 6.6	60.9 ± 5.7	0.188*
DAP (mmHg)	24.6 ± 3.0	23.8 ± 2.9	0.375*	34.0 ± 5.9	35.5 ± 4.6	0.343*
MAP (mmHg)	31.9 ± 3.0	31.1 ± 3.1	0.405*	42.0 ± 5.8	44.0 ± 4.6	0.202*
SaO_2_ (%)	93.6 ± 1.9	93.0 ± 2.3	0.386*	85.1 ± 1.6	81.7 ± 1.8	**<0.001** *
SaO_2_ – rSO_2_s (%)	24.1 ± 3.9	24.1 ± 5.9	0.987*	18.2 ± 3.3	15.0 ± 5.5	**0.031** *
SaO_2_ – rSO_2_c (%)	23.0 ± 3.6	22.8 ± 5.7	0.910**	16.0 (15.0–18.0)	11.5 (10.0–16.0)	**0.005** **
Creatinine levels (mg/dL)	0.9 ± 0.6	0.9 ± 0.6	0.900*	0.6 (0.4–1.4)	0.6 (0.4–0.6)	0.376**
Decrease in post-biPAB etCO_2_ (day 0) (mmHg)				2.3 ± 0.7	3.9 ± 0.8	**<0.001** *
Decrease in post-biPAB etCO_2_ (day 3) (mmHg)				4.1 ± 0.9	6.2 ± 0.9	**<0.001** *
Flow acceleration in RPA after biPAB (day 3) (m/s)				2.5 ± 0.3	3.2 ± 0.2	**<0.001** *
Flow acceleration in LPA after biPAB (day 3) (m/s)				2.5 ± 0.2	3.0 ± 0.3	**<0.001** *

* Independent-samples *t*-test.

** Mann–Whitney *U*-test. Bold indicates significance level at *p* value < 0.05.

biPAB, bilateral pulmonary artery banding; DAP, diastolic arterial pressure; etCO_2_, end-tidal carbon dioxide; LPA, left pulmonary artery; MAP, mean arterial pressure; RPA, right pulmonary artery; rSO_2_s, somatic regional tissue oxygen saturation; rSO_2_c, cerebral regional tissue oxygen saturation; SAP, systolic arterial pressure; SaO_2_, oxygen saturation.

Values are presented as mean ± standard deviation or median (interquartile range), as appropriate. *p*-values represent between-group comparisons at each time point. Day 0 values represent immediate post-banding measurements, whereas day 3 values correspond to postoperative day 3 assessments. Systemic hemodynamic and physiological parameters before and after bilateral pulmonary artery banding according to banding strategy. This table summarizes global hemodynamic changes.

**Table 4:** Baseline and early postoperative physiological parameters according to banding strategy.

Parameter	Conventional (*n* = 20) Mean ± SD	Physiology-guided (*n* = 24) Mean ± SD	*p*-value
Preoperative (before biPAB)			
Age at biPAB (days)	9.1 ± 5.6	9.3 ± 5.1	0.910*
Body weight (g)	2,806.5 ± 303.8	2,669.6 ± 407.6	0.221*
Pre-biPAB Saturation (%)	93.6 ± 1.8	93.0 ± 2.3	0.386*
pH before biPAB	7.2 (7.1–7.3)	7.3 (7.2–7.3)	0.741**
Lactate before biPAB (mmol/L)	3.3 (2.8–5.6)	3.4 (2.5–6.4)	0.906**
SAP before biPAB (mmHg)	46.7 ± 4.7	45.2 ± 4.6	0.285*
DAP before biPAB (mmHg)	24.5 ± 3.0	23.7 ± 2.9	0.375*
MAP before biPAB (mmHg)	31.9 ± 2.9	31.1 ± 3.1	0.405*
rSo2s before biPAB (%)	69.5 ± 2.7	68.9 ± 5.1	0.646*
rSo2c before biPAB (%)	70.6 (69.0–72.7)	70.2 (67.2–74.0)	0.750**
Sao2 - rSo2s before biPAB (%)	24.1 ± 3.9	24.1 ± 5.9	0.987*
Sao2 - rSo2c before biPAB (%)	23.0 ± 3.6	22.8 ± 5.7	0.910*
Creatinine before biPAB (mg/dL)	0.9 ± 0.6	0.9 ± 0.6	0.900*
Immediate postoperative (Day 0)			
Post-biPAB saturation (%)	85.1 ± 1.6	81.7 ± 1.8	**<0.001** *
Decrease in post-biPAB etCO_2_ (Day 0) (mmHg)	2.3 ± 0.7	3.9 ± 0.8	**<0.001** *
SAP after biPAB (mmHg)	58.4 ± 6.5	60.9 ± 5.7	0.188*
DAP after biPAB (mmHg)	33.9 ± 5.8	35.5 ± 4.6	0.343*
MAP after biPAB (mmHg)	42.0 ± 5.8	44.0 ± 4.6	0.202*
rSo2s after biPAB (%)	66.9 ± 2.9	67.1 ± 4.4	0.880*
rSo2c after biPAB (%)	69.0 (69.0–72.7)	70.0 (67.2–74.0)	0.343**
Sao2 - rSo2s after biPAB (%)	18.1 ± 3.3	15.0 ± 5.5	**0.031** *
Sao2 - rSo2c after biPAB (%)	16.0 (15.0–18.0)	11.5 (10.0–16.0)	**0.005** **
Creatinine after biPAB (mg/dL)	0.6 (0.4–1.4)	0.6 (0.4–0.6)	0.376**
Early postoperative (Day 3)			
Decrease in post-biPAB etCO_2_ (Day 3) (mmHg)	4.1 ± 0.8	6.2 ± 0.9	**<0.001** *
Flow acceleration in RPA (Day 3) (m/s)	2.5 ± 0.2	3.1 ± 0.2	**<0.001** *
Flow acceleration in LPA (Day 3) (m/s)	2.5 ± 0.2	3.0 ± 0.3	**<0.001**

* Independent-samples *t*-test.

** Mann–Whitney *U*-test. Bold indicates significance level at *P* value < 0.05.

biPAB, bilateral pulmonary artery banding; DAP, diastolic arterial pressure; etCO₂, end-tidal carbon dioxide; LPA, left pulmonary artery; MAP, mean arterial pressure; RPA, right pulmonary artery; rSO₂c, cerebral regional oxygen saturation; rSO₂s, somatic regional oxygen saturation; SAP, systolic arterial pressure; SaO₂, arterial oxygen saturation.

Values are presented as mean ± standard deviation or median (interquartile range), as appropriate. *p*-values represent between-group comparisons at each time point.

### Pulmonary blood flow modulation

**Figure 4:**
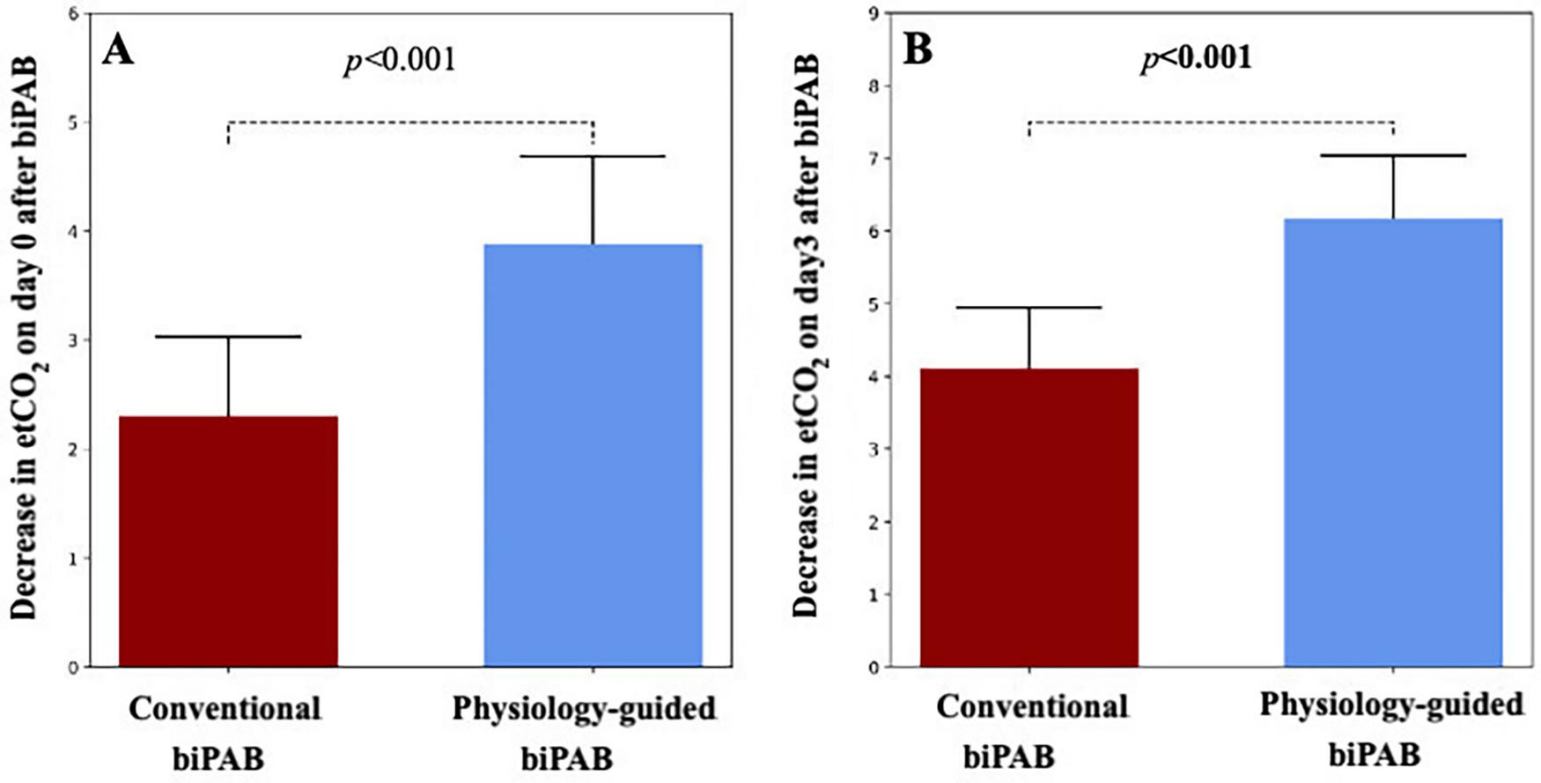
Changes in end-tidal carbon dioxide (etCO₂) following bilateral pulmonary artery banding. Panel **A** shows immediate postoperative changes, and Panel **B** shows values on postoperative day 3. Differences between conventional and physiology-guided banding strategies at both time points are indicated.

Markers of pulmonary blood flow differed between the two banding strategies. The physiology-guided group demonstrated greater reductions in end-tidal carbon dioxide (etCO₂) immediately following banding and on postoperative day 3 compared with the conventional group. These differences are shown in Figure 4.

Echocardiographic assessment demonstrated higher and more consistent Doppler-derived flow accelerations across patients in the physiology-guided group, indicating reduced inter-patient variability in band-related flow restriction. Flow velocities exceeding 3.0 m/s were more frequently observed in this group, whereas lower and more variable velocities were observed in the conventional banding group. Comparative echocardiographic and physiological parameters between strategies are detailed in Table 4.

### Oxygenation and end-organ perfusion

Postoperative arterial oxygen saturation values were lower in the physiology-guided group. Despite this reduction, the differences between arterial oxygen saturation and regional cerebral and somatic oxygenation were smaller in the physiology-guided group.

Absolute cerebral and somatic regional oxygen saturation (rSO₂) values remained stable and comparable between groups throughout the early postoperative period, without evidence of deterioration in regional oxygenation. Regional perfusion trends derived from near-infrared spectroscopy are summarized in Table 4.

### Clinical trajectory after biPAB

Following emergency bilateral pulmonary artery banding, patients demonstrated heterogeneous clinical trajectories reflecting underlying anatomy and physiological reserve. Ten patients were ultimately managed toward biventricular repair, while 34 patients followed a single-ventricle palliative pathway.

Individual patient-level characteristics, banding strategies, subsequent surgical trajectory, and clinical outcomes are summarized in Table 5. Early postoperative outcomes, interstage mortality, and subsequent clinical trajectories following bilateral pulmonary artery banding are summarized in Table 6.

**Table 5:** Individual patient characteristics, banding strategy, and subsequent surgical trajectory following bilateral pulmonary artery banding.

PN	Diagnosis	biPAB age (days)	biPAB BW (g)	PDA stent	Banding Strategy	Second surgery	Third surgery	Current status
1	TGA-large VSD	7	2800	None	Conventional	Jatene	-	Alive
2	HLHS (MS-AS), AH	6	2600	None	Physiology-guided	Delayed S1P, Sano	S2P	Awaiting Fontan
3	HLHS (MA-AA), AH	4	3500	None	Conventional	Delayed S1P, Sano	S2P	Awaiting Fontan
4	CAVCD, AH, BAV	27	2300	None	Conventional	-	-	Deathafter biPAB
5	HLHS (MA-AS), AH	3	2500	None	Physiology-guided	Delayed S1P, Sano	-	Awaiting S2P
6	HLHS (MA-AA)	6	3300	None	Conventional	Delayed S1P, Sano	-	Awaiting S2P
7	HLHS (MA-AA)	7	2500	None	Physiology-guided	Comprehensive S2P	-	Awaiting Fontan
8	SV type large VSD, AH	18	3100	None	Physiology-guided	Arch repair, MPA banding	S2P	Awaiting Fontan
9	HLHS (AA), CAVCD (unbalanced)	6	2600	None	Conventional	Delayed S1P, MBT	S2P	Awaiting Fontan
10	HLHS (MS-AS)	7	3100	None	Physiology-guide	-	-	Death after biPAB
11	BAV, AH, VSD	10	2900	None	Conventional	-	-	Death after biPAB
12	DILV, DTGA, AH	10	3800	None	Physiology-guided	Arch repair,MPA banding	S1P-BVFe	Fontan
13	Borderline LVH, AH	25	3000	None	Physiology-guided	Arch repair, debanding, ASD closure	-	Alive
14	HLHS (MA-AS)	12	3200	None	Physiology-guided	-	-	Death after biPAB
15	HLHS (MA-AA)	10	3000	None	Conventional	-	-	Death after biPAB
16	HLHS (MA-AA), aortic interruption	5	2500	None	Physiology-guided	Delayed S1P, Sano	-	Interstage death
17	HLHS (MA-AA), AH	10	3000	Yes	Physiology-guided	Delayed S1P, MBT		Interstage death
18	HLHS (MA-AA), AH	10	3200	Yes	Conventional	Delayed S1P, Sano	S2P	Fontan
19	HLHS (MA-AA)	9	2800	None	Conventional	Delayed S1P, Sano	S2P	Fontan
20	HLHS (MS-AA), AH	21	2300	None	Conventional	Delayed S1P, MBT	S2P	Fontan
21	DILV, DTGA, AH, RVH	17	3200	None	Physiology-guided	Delayed S1P, MBT	S2P	Awaiting Fontan
22	HLHS (MS-AS)	12	2750	None	Conventional	Delayed S1P, MBT	S2P	Awaiting Fontan
23	HLHS (MA-AA), AH	6	2800	None	Physiology-guided	Delayed S1P, MBT	S2P	Awaiting Fontan
24	HLHS (MA-AS), AH	8	2600	None	Physiology-guided	-	-	Death after biPAB
25	HLHS (MA-AA), AH	11	2500	None	Physiology-guided	Delayed S1P, Sano	-	Awaiting S2P
26	DILV, DTGA, AH, RVH	7	2750	None	Conventional	Delayed S1P, Sano	-	Awaiting S2P
27	SV type large VSD, AH	4	2800	None	Conventional	Delayed S1P, MBT	-	Interstage death
28	CAVCD, AH, BAV	6	2250	None	Physiology-guided	CAVCD complete correction	-	Alive
2	HLHS (MA-AA), aortic interruption	6	2500	None	Physiology-guided	Delayed S1P, Sano	-	Awaiting S2P
30	HLHS (MA-AA)	10	2650	None	Physiology-guided	Delayed S1P, Sano	S2P	Awaiting Fontan
31	DILV, DTGA, AH	8	2750	None	Conventional	-	-	Death after biPAB
32	HLHS (MA-AS)	6	2790	None	Conventional	-	-	Death after biPAB
33	TGA-large VSD	11	2570	None	Conventional	-	-	Death after biPAB
34	Borderline LVH, AH	12	2630	None	Physiology-guided	Arch repair, debanding, ASD closure	-	Alive
35	HLHS (MS-AS)	10	2800	None	Physiology-guided	Delayed S1P, Sano	S2P	Awaiting Fontan
36	HLHS (AA), CAVCD (unbalanced)	13	2640	None	Physiology-guided	Delayed S1P, Sano	-	Awaiting S2P
37	HLHS (MA-AA), AH	7	2900	None	Conventional	-	-	Death after biPAB
38	HLHS (MA-AA), aortic interruption	5	3000	None	Conventional	Delayed S1P, Sano	S2P	Awaiting Fontan
39	BAV, AH, VSD	5	2700	None	Conventional	Arch repair, MPA banding	-	Alive
40	HLHS (MA-AA)	4	2600	None	Physiology-guided	Delayed S1P, Sano	-	Awaiting S2P
41	BAV, AH, VSD	7	2200	None	Physiology-guided	Arch repair, MPA banding	Debanding	Alive
42	HLHS (MA-AA)	6	2300	None	Physiology-guided	Delayed S1P, Sano	-	Interstage death
43	CAVCD, AH, BAV	8	2420	None	Conventional	-	-	Death after biPAB
44	HLHS (MA-AS)	5	2100	None	Physiology-guided	Delayed S1P, MBT	S2P	Awaiting Fontan

AA, aortic atresia; AH, aortic arch hypoplasia; AS, aortic stenosis; ASO, arterial switch operation; BAV, bicuspid aortic valve; biPAB, bilateral pulmonary artery banding; BVFe, bulboventricular foramen enlargement; BVR, biventricular repair; BW, body weight; CAVCD, complete atrioventricular canal defect; DILV, double inlet left ventricle; HLHS, hypoplastic left heart syndrome; LVH, left ventricular hypoplasia; MBT, modified Blalock–Taussig shunt; MPA, main pulmonary artery; PDA, patent ductus arteriosus; RV, right ventricle; S1P, stage I palliation; S2P, stage II palliation; SV, single ventricle; TGA, transposition of the great arteries; VSD, ventricular septal defect.

**Table 6:** Early postoperative outcomes, interstage mortality, and clinical trajectories following bilateral pulmonary artery banding according to banding strategy.

Outcome and status	Conventional biPAB (*n* = 20)	Physiology-guided biPAB (*n* = 24)
Mortality		
Early postoperative	5	2
Interstage	4	4
Survivors		
Single-ventricle pathway	9	14
Biventricular pathway	2	4
Awaiting next stage		
Awaiting Stage I palliation	0	0
Awaiting Stage II palliation	2	5
Awaiting Stage III palliation	4	8
Completed Stage III palliation	3	1

biPAB, bilateral pulmonary artery banding.

Values represent the number of patients in each category.

### Survival and mortality

Following bilateral pulmonary artery banding, 33 of 44 high-risk neonates survived the immediate postoperative period, corresponding to an early survival rate of 75%. During follow-up, additional interstage mortality occurred exclusively within the single-ventricle pathway. At last follow-up, 29 patients remained alive, yielding a cumulative survival rate of 66%.

These survival proportions and patient trajectories across the study cohort are summarized in Figure 3.

### Mortality characteristics and non-significant findings

All mortality events occurred in the context of persistent sepsis, multiorgan dysfunction, or advanced physiological compromise at presentation. No deaths were directly attributable to pulmonary artery band–related complications, excessive pulmonary blood flow restriction, band migration, or acute band-related hemodynamic collapse.

No statistically significant between-group differences were observed with respect to age at banding, baseline arterial blood gas parameters, lactate levels, baseline regional oxygenation values, presence of sepsis or shock, genetic syndromes, or overall survival status.

## Discussion

The present study suggests that a physiology-guided approach to bilateral pulmonary artery banding (biPAB) in high-risk neonates was associated with more consistent modulation of pulmonary blood flow and more favorable early hemodynamic and perfusion profiles compared with a conventional uniform banding strategy (1, 2, 12). Importantly, these physiological differences were observed without an apparent increase in early postoperative or interstage mortality, despite the inclusion of a higher proportion of low-birth-weight neonates and patients with significant preoperative instability in the physiology-guided group (13, 14).

### Physiology-guided banding and pulmonary blood flow control

Effective regulation of pulmonary blood flow represents a central determinant of successful biPAB, particularly in neonates with ductal-dependent circulation, in whom small alterations in band tightness or pulmonary vascular resistance may lead to disproportionate shifts in systemic and pulmonary perfusion (15, 30, 31). In this cohort, physiology-guided banding was associated with greater and more consistent reductions in end-tidal carbon dioxide (etCO₂), together with higher and less variable Doppler-derived pulmonary artery flow velocities, with reduced interpatient variability**.** When interpreted collectively, these findings suggest a more uniform physiological response to pulmonary blood flow restriction across a heterogeneous population (32–34). Efforts were made to minimize measurement variability using standardized intraoperative marking techniques, thereby improving the practical reproducibility of small adjustments in band circumference.

Uniform band sizing applies similar anatomical constraints across a wide range of body weights and pulmonary artery dimensions. In contrast, weight-adjusted, z-score–guided banding explicitly accounts for interpatient variability (7, 8, 11). Although the present study was not designed to establish causality, the observed consistency of pulmonary flow markers supports the concept that individualized banding strategies may reduce variability in early postoperative physiology, particularly in physiologically fragile neonates (9). Accordingly, the present findings should be interpreted as reflecting differences in physiological response rather than direct quantitative differences in achieved band tightness.

Recent studies have increasingly emphasized the importance of individualized and physiology-guided strategies in neonatal circulatory management, supporting the rationale for tailored approaches to pulmonary blood flow modulation (26–29, 35, 36). The physiology-guided approach may be particularly beneficial in patients with highly labile circulatory physiology, such as those with ductal-dependent systemic or pulmonary circulation, where small changes in pulmonary blood flow may result in disproportionate hemodynamic effects.

Representative target-sizing examples demonstrated that the physiology-guided strategy generally produced smaller target branch pulmonary artery diameters than the conventional 3.0-mm approach in lower-weight neonates, particularly in the 2,000–2,500 g range. This difference became negligible at approximately 2,750 g and slightly reversed at higher weights.

**Figure 5:**
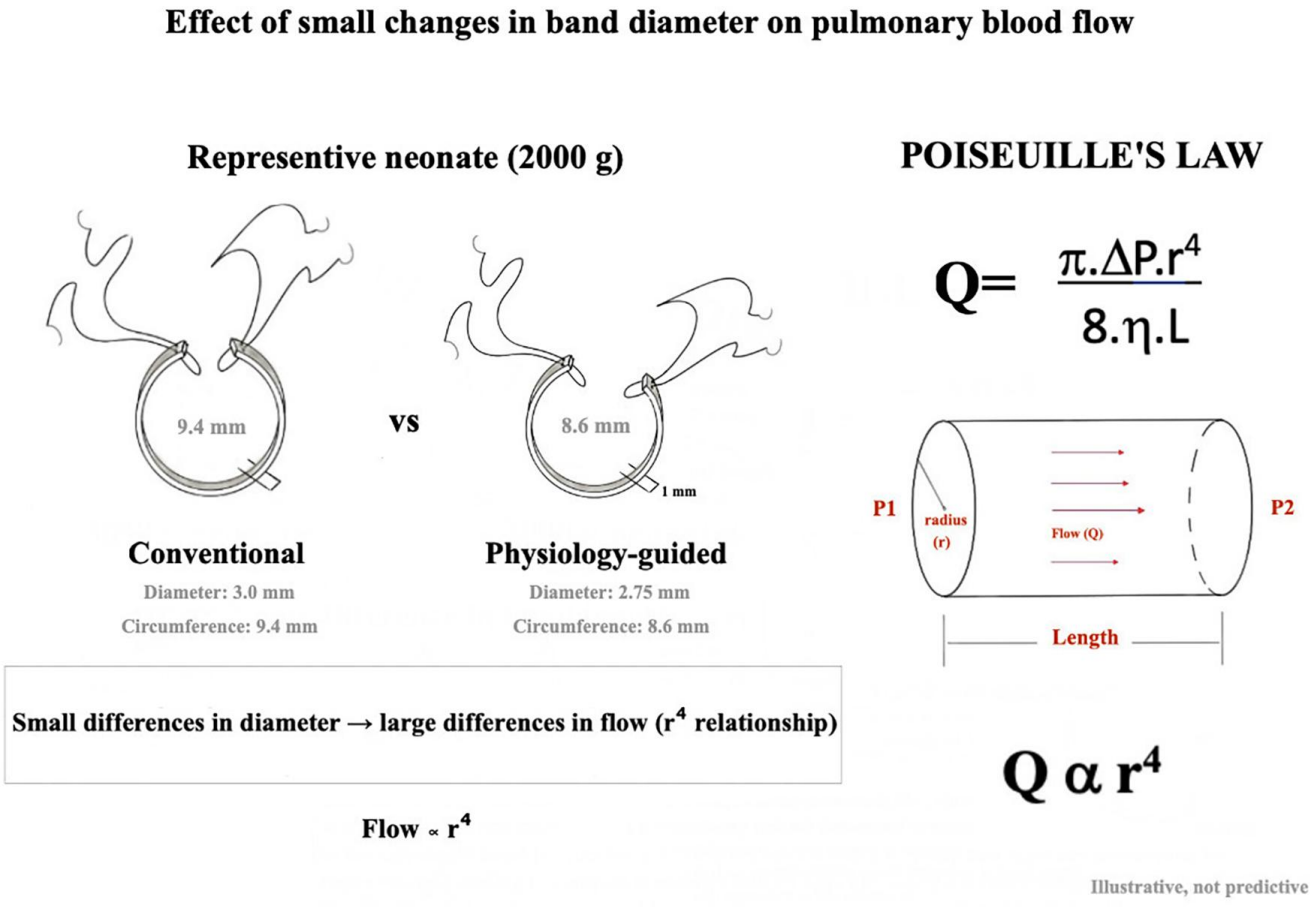
Comparison of conventional and physiology-guided pulmonary artery band sizing in a representative 2000g neonate. For the same body weight, conventional banding applies a fixed diameter (3.0 mm), whereas the physiology-guided strategy targets a smaller diameter corresponding to approximately −2 z-score (≈2.75 mm), resulting in a difference in band circumference (≈9.4 mm vs. ≈8.6 mm). This figure is intended for conceptual illustration.

Although these differences are numerically small, they may be physiologically significant. According to Poiseuille's law, flow is proportional to the fourth power of vessel radius. For example, the difference between a circumference of approximately 8.6 mm and 9.4 mm theoretically corresponds to an increase in flow of approximately 50%–60%. These findings highlight how small millimetric differences in band diameter may translate into substantial changes in pulmonary blood flow, as demonstrated in Figure 5.

### Interpretation of etCO₂ and multimodal physiological monitoring

The interpretation of etCO₂ as a surrogate marker of pulmonary blood flow warrants particular attention. In mechanically ventilated neonates without significant ventilation–perfusion mismatch, changes in etCO₂ predominantly reflect alterations in pulmonary perfusion and cardiac output (3, 4, 10). The greater reductions in etCO₂ observed in the physiology-guided group are therefore consistent with greater attenuation of pulmonary overcirculation.

Crucially, etCO₂ was not interpreted in isolation but rather integrated with systemic arterial pressure, echocardiographic findings, and regional oxygenation data. This multimodal monitoring framework reduces the risk of misinterpretation and highlights the value of combining anatomical planning with real-time physiological feedback during pulmonary artery banding (5, 6, 37). Such integration is particularly relevant in neonates, in whom small anatomical differences may result in large physiological consequences.

### End-organ perfusion and oxygenation balance

Lower postoperative arterial oxygen saturation observed in the physiology-guided group should be interpreted as an expected physiological consequence of effective pulmonary blood flow restriction rather than as an adverse outcome (16). In ductal-dependent neonates, modest reductions in arterial saturation often accompany improved systemic perfusion pressure and redistribution of cardiac output toward systemic circulation (17).

Notably, the physiology-guided group demonstrated smaller differences between arterial oxygen saturation and regional cerebral and somatic oxygenation. This pattern suggests improved coupling between systemic oxygen delivery and tissue-level oxygen utilization (19–21). Importantly, absolute regional oxygenation values remained stable, indicating that this apparent improvement in balance was achieved without compromising cerebral or somatic perfusion.

### Clinical implications in low-birth-weight and borderline patients

Low birth weight is a well-established risk factor for adverse outcomes following neonatal cardiac surgery (18, 22). In the present study, the physiology-guided group included a significantly higher proportion of low-birth-weight neonates yet demonstrated comparable or more favorable early physiological profiles. This observation suggests that individualized banding strategies may be especially informative in smaller patients, in whom millimeter-level differences in band circumference can translate into substantial physiological effects (23). This concept is illustrated in Figure 5, which demonstrates how small differences in band diameter may translate into substantial differences in pulmonary blood flow due to the nonlinear relationship between vessel radius and flow.

In addition, biPAB functioned as a valuable bridging strategy in patients with borderline ventricular morphology. By stabilizing hemodynamics and controlling pulmonary blood flow, physiology-guided biPAB allowed time for serial ventricular reassessment and informed decision-making regarding definitive surgical pathways (24, 38, 39). This intentional period of physiological stabilization may help avoid premature commitment to a single-ventricle or biventricular strategy in anatomically and functionally indeterminate patients.

### Ductal patency strategy and interstage management

Maintenance of ductal patency represents an integral component of hybrid palliation strategies (40, 41). In the present cohort, continuous prostaglandin E1 infusion was preferentially used, with ductal stenting reserved for cases of prostaglandin-resistant ductal constriction. This approach was guided by concerns that ductal stenting in the presence of severe aortic arch hypoplasia or ductal-dependent coronary circulation may adversely affect retrograde aortic and coronary perfusion (25, 42).

Prolonged prostaglandin therapy provided a stable and predictable means of maintaining ductal patency without introducing additional anatomical variables during a period of marked physiological vulnerability. The low requirement for ductal stenting and absence of prostaglandin-related complications support the feasibility of this strategy in carefully selected patients (43).

### Clinical outcomes and surgical trajectory

Early postoperative and interstage mortality did not differ significantly between banding strategies and was primarily associated with persistent sepsis, multiorgan dysfunction, or delayed referral rather than with band-related hemodynamic instability (44). These findings suggest that physiology-guided banding does not compromise clinical safety while offering potential physiological advantages.

Progression through staged palliation or toward biventricular repair was feasible in the majority of surviving patients. Although the present study was not powered to detect differences in long-term outcomes, improved early physiological stability may influence subsequent surgical planning and interstage management (45).

### Positioning within the hybrid palliation literature

Hybrid strategies combining bilateral pulmonary artery banding with maintenance of ductal patency have become established alternatives to primary neonatal cardiopulmonary bypass in selected high-risk patients (35, 40, 46). However, substantial heterogeneity exists in reported banding techniques, physiological targets, and outcome measures. Many prior studies emphasize survival or stage progression, with comparatively limited focus on the physiological mechanisms underlying early postoperative stability (36, 47).

By emphasizing physiological and perfusion-related endpoints, the present study contributes mechanistic insight into how pulmonary blood flow modulation influences systemic perfusion in the immediate postoperative period. These findings support the concept that pulmonary artery banding should be viewed as a dynamic, physiology-responsive intervention rather than a fixed anatomical procedure.

Randomized trials comparing banding strategies in critically ill neonates are unlikely to be feasible. Within this constraint, carefully conducted observational studies incorporating detailed physiological characterization remain essential. In this context, the present analysis provides incremental but clinically relevant evidence supporting the integration of individualized, physiology-guided strategies and multimodal monitoring into neonatal pulmonary artery banding.

### Limitations

Several limitations of this study should be acknowledged. Because the physiology-guided strategy was progressively adopted during the study period, potential confounding related to operator experience, perioperative learning effects, and temporal improvements in postoperative and interstage management cannot be completely excluded, despite the partial overlap between strategies. In addition, actual achieved band diameters or circumferences were not systematically recorded, precluding direct quantitative comparison of final band tightness between strategies. The retrospective design precludes definitive causal inference and may be subject to selection bias, despite the use of predefined institutional practices guiding banding strategy. The study population was heterogeneous, encompassing a wide spectrum of congenital cardiac anatomies and physiological states, which limited the feasibility of meaningful subgroup analyses. Patients requiring extracorporeal support were excluded, potentially underrepresenting the most critically unstable neonates and limiting generalizability to this subgroup. The sample size was modest, and observed associations between physiological markers should therefore be interpreted as exploratory. In addition, long-term outcomes, including pulmonary artery growth, ventricular remodeling, and neurodevelopmental status, were beyond the scope of this analysis. Accordingly, these findings should be regarded as hypothesis-generating rather than definitive. Survival proportions are reported descriptively and were not derived from formal time-to-event analyses. Pulmonary artery wall thickness was not systematically assessed, as echocardiographic evaluation was primarily focused on luminal diameters. Therefore, band sizing was based on luminal measurements and physiological response rather than vessel wall characteristics.

### Future directions

Prospective studies incorporating predefined physiological targets, standardized monitoring protocols, and longer-term follow-up are warranted to further refine pulmonary artery banding strategies in high-risk neonates. In particular, future investigations should aim to evaluate the impact of physiology-guided approaches on pulmonary artery growth, ventricular remodeling, and neurodevelopmental outcomes. The integration of advanced physiological monitoring, imaging-based flow assessment, and computational modeling may further support individualized surgical planning and improve reproducibility in this vulnerable population.

## Conclusions

In high-risk neonates with ductal-dependent circulation, a physiology-guided approach to bilateral pulmonary artery banding using weight-adjusted pulmonary artery z-scores was associated with more predictable modulation of pulmonary blood flow and more consistent early hemodynamic and perfusion profiles. These physiological advantages were observed without an apparent increase in early or interstage mortality. Within the limitations of a retrospective design, these findings support the feasibility of incorporating individualized, physiology-driven principles and multimodal physiological monitoring into neonatal pulmonary artery banding strategies. Moreover, biPAB may serve not only as a stabilizing intervention but also as a decision-enabling strategy in critically ill neonates, providing time for physiological recovery and informed surgical planning.

## Data Availability

The original contributions presented in the study are included in the article/Supplementary Material, further inquiries can be directed to the corresponding author.
